# Increase in cerebral oxygenation during advanced life support in out-of-hospital patients is associated with return of spontaneous circulation

**DOI:** 10.1186/s13054-015-0837-5

**Published:** 2015-03-24

**Authors:** Cornelia Genbrugge, Ingrid Meex, Willem Boer, Frank Jans, René Heylen, Bert Ferdinande, Jo Dens, Cathy De Deyne

**Affiliations:** Faculty of Medicine and Life Sciences, Hasselt University, Martelarenlaan 42, 3500 Hasselt, Belgium; Department of Anesthesiology, Intensive Care, Emergency Medicine and Pain Therapy, Ziekenhuis Oost-Limburg Genk, Schiepse Bos 6, 3600 Genk, Belgium; Department of Cardiology, Ziekenhuis Oost-Limburg, Schiepse Bos 6, 3600 Genk, Belgium

## Abstract

**Introduction:**

By maintaining sufficient cerebral blood flow and oxygenation, the goal of cardiopulmonary resuscitation (CPR) is to preserve the pre-arrest neurological state. To date, cerebral monitoring abilities during CPR have been limited. Therefore, we investigated the time-course of cerebral oxygen saturation values (rSO_2_) during advanced life support in out-of-hospital cardiac arrest. Our primary aim was to compare rSO_2_ values during advanced life support from patients with return of spontaneous circulation (ROSC) to patients who did not achieve ROSC.

**Methods:**

We performed an observational study to measure rSO_2_ using Equanox™ (Nonin, Plymouth, MI) from the start of advanced life support in the pre-hospital setting.

**Results:**

rSO_2_ of 49 consecutive out-of-hospital cardiac arrest patients were analyzed. The total increase from initial rSO_2_ value until two minutes before ROSC or end of advanced life support efforts was significantly larger in the group with ROSC 16% (9 to 36) compared to the patients without ROSC 10% (4 to 15) (*P* = 0.02). Mean rSO_2_ from the start of measurement until two minutes before ROSC or until termination of advanced life support was higher in patients with ROSC than in those without, namely 39% ± 7 and 31% ± 4 (*P* = 0.05) respectively.

**Conclusions:**

During pre-hospital advanced life support, higher increases in rSO_2_ are observed in patients attaining ROSC, even before ROSC was clinically determined. Our findings suggest that rSO_2_ could be used in the future to guide patient tailored treatment during cardiac arrest and could therefore be a surrogate marker of the systemic oxygenation state of the patient.

## Introduction

The goal of cardiopulmonary resuscitation (CPR) is to preserve the pre-arrest neurological state by maintaining sufficient cerebral blood flow and oxygenation. By generating sufficient oxygenated blood flow, ischemic brain damage is minimized [1]. However, only a minority of patients following out-of hospital cardiac arrest (OHCA) experience survival without neurological injury [2-4]. Good quality bystander basic life support, shorter time between collapse and start of CPR and early defibrillation increase the chance of survival with good neurological outcome [5]. Unfortunately, it is still impossible to obtain any information about organ perfusion, in particular cerebral perfusion, and ensuing ischemic organ damage during CPR. Current monitoring of the OHCA patient during CPR is limited to rhythm assessments, pulse checks between two episodes of chest compressions and end-tidal carbon dioxide [6]. Currently, the latter is the only parameter proven to correlate with the likelihood of return of spontaneous circulation (ROSC), although its value for the prediction of long-term outcome has not been established and gives no particular information about the brain [7].

An ideal monitor during out-of hospital CPR should be readily available for use, non-invasively, independent of a pulsatile signal and should provide information about the condition of the brain. Cerebral near-infrared spectroscopy (NIRS), a non-invasive technique, measures the regional difference between oxygenated and deoxygenated hemoglobin, an expression of the difference in oxygen supply and demand. NIRS is independent of a pulsatile signal and continuously measures cerebral oxygen saturation (rSO_2_) [8]. This technique has recently been introduced in the cardiac arrest setting [9-17]. These studies suggest that rSO_2_ measured during CPR may correlate with ROSC and survival. Unfortunately, all currently available data were obtained during in-hospital CPR efforts of OHCA patients or during CPR of in-hospital cardiac arrest patients. Until now, no data are available on rSO_2_ monitoring from start of advanced life support (ALS) in patients suffering from OHCA. The purpose of this study was to measure rSO_2_ in OHCA patients from the start of ALS until ROSC or until the resuscitation efforts were terminated. We aimed to investigate whether any difference existed in the time course of rSO_2_ between patients who did or did not experience ROSC.

## Methods

In this prospective, observational, single-center study (Ziekenhuis Oost-Limburg, Genk, Belgium) we measured pre-hospital rSO_2_ in consecutive OHCA patients. The primary aim of this study was to investigate whether there was any difference in rSO_2_ during OHCA ALS between patients who achieved ROSC and patients who do not. Furthermore, the difference in highest, lowest and initial rSO_2_ was also investigated as the proportion of time during ALS spent below 30%. The study protocol was approved by the local institutional review board (Commissie Medische Ethiek Ziekenhuis Oost-Limburg, 13–044 U). Informed consent was obtained from patient’s next of kin. In circumstances in which it was not possible to obtain informed consent, the requirement for informed consent was waived, in agreement with the protocol.

An emergency vehicle, staffed by a physician trained in emergency medicine or anesthesiology and a nurse, equipped with a portable cerebral oximeter (Equanox™ 7600; Nonin Medical Inc., Plymouth, MN, USA) drove to the scene of an OHCA after an emergency call. The physician placed a single sensor on the right side of the forehead of the patient as soon as possible, in some cases immediately and in some cases after endotracheal intubation. If the sensor was applied before endotracheal intubation, 100% oxygen was delivered through a bag valve mask. All patients were intubated and ventilated with 100% oxygen. Only one rSO_2_ sensor was applied to minimize the time delay to the gold standard of care. rSO_2_ was continuously measured and registered every 4 seconds during pre-hospital ALS until CPR efforts were terminated or until the patient with ROSC arrived at the emergency department. ALS was performed following current European Resuscitation guidelines [6]. In all patients, manual chest compressions were performed. None of the patients received mechanical chest compressions. If ROSC was achieved, the emergency physician pressed the event button of the cerebral oximeter and/or noted the time of ROSC on the Utstein forms [18,19]. Patients were pronounced dead pre hospital or they achieved sustained ROSC (>20 minutes) and were transported to the hospital. No patient was transported with ongoing CPR.

Exclusion criteria were age younger than 18, obvious traumatic cause of cardiac arrest and ROSC before or within 1 minute after start of rSO_2_ measurement. Emergency physicians were not blinded to rSO_2_ values because visual confirmation of the measurement and quality of the NIRS signal is necessary. However, they had not received any pre-study information regarding the interpretation of rSO_2_ values. The rSO_2_ values were therefore not used in the clinical decision-making and all patients received the best available care regardless of rSO_2_ values. The decision to stop resuscitation efforts was at the discretion of the medical emergency team. The accuracy and reliability of the used cerebral oximeter (Equanox™ 7600; Nonin Medical Inc.) has been validated against a mix of 70% jugular bulb saturation and 30% arterial saturation [20].

Baseline characteristics were prospectively collected from Utstein templates and emergency medical charts [18,19]. rSO_2_ data were downloaded from the portable device according to the manufacturer’s instructions and exported to SPSS 20.00 (SPSS, Armonk, NY, USA) for statistical analysis. Patients were defined as achieving ROSC if ROSC was sustained for more than 20 minutes.

Because the goal of this prospective study was to investigate the rSO_2_ values in OHCA patients during ALS as a prediction for ROSC and to avoid interference from rSO_2_ measurements when ROSC was already achieved, only data obtained until 1 or 2 minutes before ROSC were used in the following data analysis. We compared initial, mean and maximum rSO_2_ values as well as the increase in rSO_2_ and the percentage of ALS time spent below a rSO_2_ value of 30%.

### Statistical analysis

Patients’ characteristics were compared using Student’s *t* test if normally distributed or the Mann–Whitney test if not normally distributed and expressed as respectively mean ± standard deviation or median with first and third quartiles. The chi-square test and Fisher’s exact test were used when comparing categorical values. Descriptive statistics were used for continuously measured rSO_2_ values and are expressed as mean ± standard deviation or median with first and third quartiles. *P* <0.05 was considered statistically significant. All tests were performed using SPSS 20.00. Figures were made using GraphPad Prism 5.01 (GraphPad Software, La Jolla, CA, USA).

## Results

**Figure 1:**
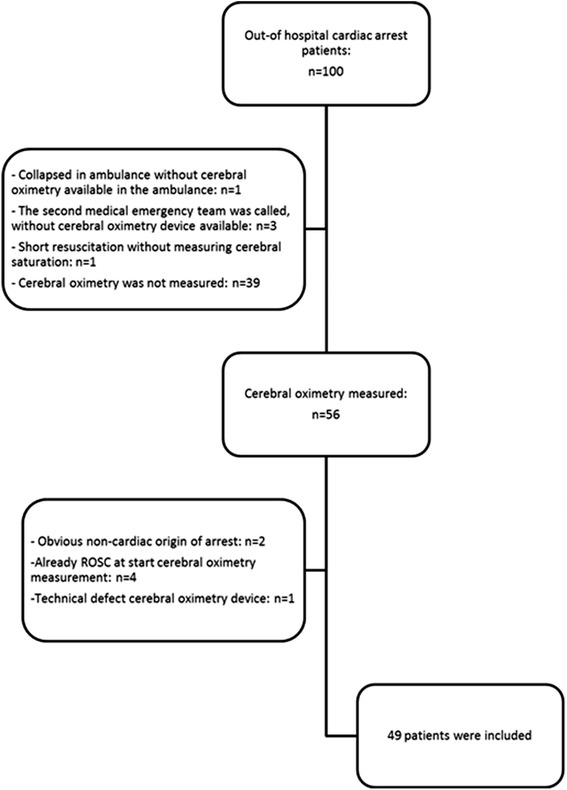
**Enrollment of study patients.** ROSC, return of spontaneous circulation.

Between December 2011 and November 2013, there were 100 eligible OHCA patients. Out of these 100 patients, rSO_2_ was measured in 56 OHCA patients (Figure 1). Data for seven patients were excluded from the analysis for the following reasons: obvious noncardiac cause of arrest (hanging) in two patients, four patients already had ROSC when rSO_2_ measurement was started, and data from one patient could not be used due to a technical defect during transfer of data. Eventually 49 patients were prospectively included, 19 (39%) patients achieved sustained ROSC whereas 30 patients (61%) did not and died pre hospital. Of the 19 patients with ROSC, 10 patients (20%) died in the emergency department and eventually three (6%) patients survived with good neurological outcome (Cerebral Performance Category 1 and 2) at hospital discharge and one patient survived with Cerebral Performance Category 4.

Comparing ROSC with no ROSC, there were significantly more male patients in the group without ROSC (77% vs. 42%, *P* = 0.03). Age, initial rhythm, witnessed arrest, amount of epinephrine given and the number of patients in which basic life support by bystanders was started were similar in both groups (Table 1). Patients with ROSC had a shorter median duration of basic life support, initiated by bystander, general practitioners or paramedics (10 (4 to 12) minutes vs. 13 (10 to 17) minutes, *P* <0.01) and shorter median duration of ALS (16 (10 to 30) minutes vs. 25 (20 to 33) minutes, *P* = 0.02). The median time between the emergency call and the start of ALS was 14 (12 to 17) minutes in the group without ROSC and 12 (8 to 15) minutes in the group with ROSC (*P* = 0.01).

**Table 1:** **Patient demographics and characteristics**

	**Total**	**No ROSC**	**ROSC**	***P*** **value**
	**(** ***n*** **= 49)**	**(** ***n*** **= 30)**	**19)**	
Male	31 (63)	23 (77)	8 (42)	0.03
Age	73 (±17)	69 (±17)	70 (16)	0.85
First recorded rhythm				
Ventricular fibrillation	12 (24)	6 (20)	6 (32)	0.50
Asystole	31 (63)	20 (67)	11 (58)	0.56
Pulseless electric activity	6 (15)	4 (13)	2 (11)	1.00
Bystander witnessed cardiac arrest	26 (53)	14 (47)	12 (63)	0.38
EMS witnessed cardiac arrest	3 (6)	1 (3)	2 (11)	0.55
Bystander CPR	29 (59)	20 (67)	9 (47)	0.24
BLS duration (minutes)				
Total	12 (9 to 16)	13 (10 to 16.75)	10 (4 to 12)	0.01
Bystander	10 (5.5 to 15)	10 (7.5 to 15)	5 (4 to 11.5)	0.02
Paramedic	6.5 (4 to 10)	8 (5 to 12)	4 (4 to 8.5)	0.03
ALS duration (minutes)	21 (15.5 to 31)	25 (20 to 32.75)	16 (10 to 30)	0.02
Response time (minutes)				
Time (call to ALS)	13 (11 to 15)	14 (12 to 17.25)	12 (8 to 15)	0.01
Time (call to BLS)	0 (0 to 4)	0 (0 to 4.75)	2 (0 to 4)	0.48
Time (ALS to sensor placement)	3 (1 to 9)	5 (1.75 to 12)	2 (0 to 6)	0.15
Epinephrine dosage (mg)	5 (3 to 8)	5 (4 to 8)	4 (2 to 8)	0.11

Data are presented as number (percentage), mean (± standard deviation) or median (interquartile range). Demographic data of studied patients. ALS, advanced life support; BLS, basic life support; CPR, cardiopulmonary resuscitation; EMS, emergency medical system; ROSC, return of spontaneous circulation.

The total median increase from initial rSO_2_ values until 2 minutes before ROSC or the end of ALS efforts was significantly larger in the group with ROSC (16% (9 to 32)) compared with the patients without ROSC 10% (4 to 15) (*P* = 0.02). All results are summarized in Table 2.

**Table 2:** **Cerebral saturation values**

	**No ROSC**	**ROSC**	***P*** **value**
	**(** ***n*** **= 30)**	**(** ***n*** **= 19)**	
Initial rSO_2_	20 % (±13)	28% (±20)	0.07
Initial rSO_2_, lowest value of the first minute	19% (7 to 30)	24% (8 to 39)	0.10
Initial rSO_2_, mean of the first minute	27% (14 to 33)	35% (8 to 43)	0.11
Lowest rSO_2_ value	19% (7 to 3)	24 %(8 to 39)	0.14
Highest rSO_2_ value, ROSC included	40% (±16)	56% (±14)	<0.01
Highest rSO_2_ until 2 minutes before ROSC	40% (±16)	52% (±14)	<0.01
Mean rSO_2_, ROSC included	31% (±4)	42% (±8)	0.02
Mean rSO_2_ until 2 minutes before ROSC	31% (±4)	39% (±7)	0.05
Increase in rSO_2_, ROSC included	10% (4 to 15)	18% (15 to 33)	<0.01
Increase in rSO_2_ until 2 minutes before ROSC	10% (4 to 15)	16% (9 to 32)	0.02
Increase in rSO_2_ until 16 minutes	11% (6 to 19) (14 pts)	16% (11 to 36) (19 pts)	0.05
% time spent rSO_2_ ≤ 30%, ROSC included	25 (0 to 100)	1 (0 to 37)	0.03
% time spent rSO_2_ ≤ 30% until 2 minutes before ROSC	25 (0 to 100)	2 (0 to 36)	0.05

Parametric data expressed as mean (± standard deviation), nonparametric data as median (interquartile range). rSO_2_, cerebral tissue saturation; ROSC, return of spontaneous circulation.

The median duration of ALS until ROSC was 16 (10 to 30) minutes. If we compare rSO_2_ from the start of measurement until ROSC or until 16 minutes in the no ROSC group (only available in 14 patients), a significant difference in median increase is observed – namely 11% (5.75 to 19) for the no ROSC group versus 16% (11 to 36) for the ROSC group (*P* = 0.05).

**Figure 2:**
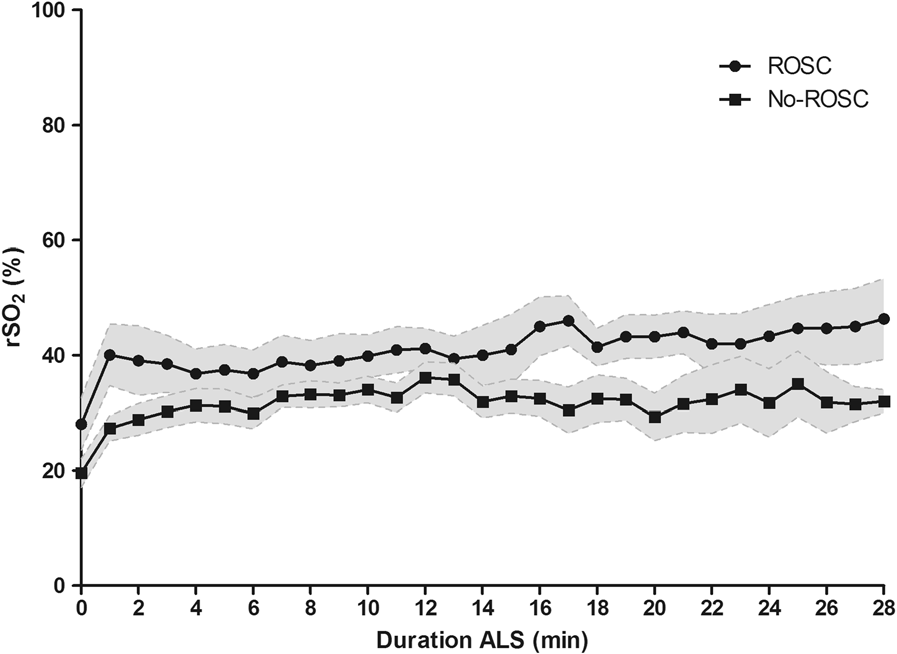
**Course of cerebral oxygen saturation during advanced life support.** Mean cerebral oxygen saturation (rSO_2_) values ± standard error of mean during advanced life support (ALS) in patients with return of spontaneous circulation (ROSC) and no ROSC.

Patients who did not achieve ROSC spent a significantly higher portion of time during ALS with rSO_2_ ≤ 30% (*P* < 0.03). The median proportion of time spent ≤30% until 2 minutes before ROSC was 25% (0 to 100) of the time for the no ROSC group compared with 2% (0 to 36) of the time for the ROSC group (*P* = 0.05). Figure 2 represents the course of rSO_2_ values in both groups during ALS.

The mean maximum rSO_2_ value until 2 minutes before ROSC recorded during ALS was significantly higher in the group that achieved ROSC (52% ± 14 vs. 40% ± 16, *P* <0.01). The median minimal rSO_2_ value recorded during ALS, 24% (8 to 39) in the ROSC group compared with 18% (7 to 29) in the no ROSC group, did not differ (*P* = 0.14) between both groups.

There was no statistically significant difference in the mean initial recorded rSO_2_ value between the ROSC group compared with the no ROSC group (28% ± 20 vs. 20% ± 13, *P* = 0.07). The mean rSO_2_ over the first minute after the start of measurement was not different between both groups, 35% (8 to 43) in the ROSC group and 27% (14 to 33) in the no ROSC group (*P* = 0.11).

The mean rSO_2_ from the start of measurement until 2 minutes before ROSC or until ALS was terminated was higher in the ROSC group compared with the no ROSC group, respectively 39% ± 7 and 31% ± 4 (*P* = 0.05).

The mean initial rSO_2_ value of the three patients with good neurological outcome was 44% (±26). The mean rSO_2_ until 2 minutes before ROSC was 54% (±4) and the increase until 2 minutes before ROSC was 13% (±6).

## Discussion

During pre-hospital ALS, a higher increase in rSO_2_ is observed in patients attaining ROSC, even before ROSC was clinically determined. rSO_2_ monitoring was started immediately on the arrival of the medical emergency team at the scene of cardiac arrest. During the entire course of ALS, rSO_2_ values were recorded, until ROSC or until the decision to stop ALS. Apart from three small feasibility studies, all previously published studies on this topic reported only data obtained in the emergency department, and therefore a considerable time after the cardiac arrest [14,17,21]. As a result of our early pre-hospital start of rSO_2_ monitoring, it was possible to monitor patients with short ALS duration and a possibly positive neurological outcome. The time delay between start of CPR and rSO_2_ monitoring in all published studies was much longer, without information on the duration of low rSO_2_ values pre hospital and with exclusion of OHCA patients with short time to ROSC [10].

As stated by others and in accordance with our results, rSO_2_ seems more useful when it is used as a dynamic information tool rather than a static one, so that a single threshold rSO_2_ value might be insufficient to predict outcome [10,11,22]. However, use of the mean rSO_2_ as the threshold is not clinically useful. We used data until 2 minutes before ROSC to enable a comparison of rSO_2_ during ALS between two equivalent groups of patients: a first group who achieved ROSC and a second group in which ALS efforts were halted, but both still in cardiac arrest. We analyzed the difference in rSO_2_ increase during ALS between patients who achieved ROSC and those who did not. We found a significant difference in the increase in rSO_2_ between these groups, with an increase in rSO_2_ even before ROSC was clinically determined by means of pulse check [23]. These observations support the idea for the use of cerebral saturation as a monitor during ALS. Asim and colleagues also analyzed the increase in rSO_2_ during CPR, and found a gradual increase of 5% (0 to 18) in the patients without ROSC compared with 52% (17 to 52) (*P* <0.001) in the patients with ROSC [16]. The higher increase, compared with our findings, could be due to the device used to measure rSO_2_, the Invos™ 5100C (Covidien, Boulder, CO, USA), which does not measure values below 15%. Secondly, the inclusion of rSO_2_ values at the moment of ROSC together with the rather small number of patients included (23 patients) will exacerbate differences between survivors and nonsurvivors. Thirdly, these findings are of limited added clinical value because ROSC was already determined.

In addition to the increase from start of rSO_2_ measurement until ROSC, a significant difference was also observed between the ROSC group and the no ROSC group if we compared the increase from start of ALS until 16 minutes of ALS (median duration of ALS in the ROSC group). Both findings could be a surrogate marker for better systemic oxygenation with ROSC as a result. These differences in the increase of rSO_2_ between the ROSC group and the no ROSC group suggest a high sensitivity of rSO_2_ to hemodynamic changes, which can be important during ALS for early determination of ROSC and rearrest, especially in the pre-hospital setting where monitoring modalities are limited in contrast to in-hospital cardiac arrest.

Analysis of the current results did not enable determination of a critical threshold of increase predicting ROSC with acceptable specificity/sensitivity. Probably, larger patient numbers will be needed and our ongoing multicenter trial (ClinicalTrials.gov NCT01806844) could enable us to analyze whether this critical threshold for rSO_2_ increase can be determined.

We observed no significant difference in initial rSO_2_ between patients who achieved ROSC and patients who did not achieve ROSC. The absence of a significant difference in initial rSO_2_ between the ROSC group and the no ROSC group was unexpected. We hypothesized that patients with shorter time between the emergency call and start of ALS would have a higher initial rSO_2_ value, which in turn is supported by a significantly shorter time between the emergency call and start of ALS in patients achieving ROSC. The findings therefore seem contradictory, but this could be linked to the relatively small patient population studied and the observed trend for higher initial rSO_2_ in the ROSC group. Initial rSO_2_ could be clinically interesting because it is a fast and easy observation. Although only rSO_2_ data during CPR were included, we were able to confirm the results of Parnia and colleagues that patients with ROSC had a majority of rSO_2_ values >30% during CPR [11]. Despite this confirmation, it is important to take into consideration that, amongst our group of survivors, initial rSO_2_ values of 0% were measured.

In a recent multicenter study, Ito and colleagues measured rSO_2_ in 672 OHCA patients during a 1-minute period at hospital arrival [15]. A significant difference between patients with transient or sustained ROSC and no ROSC was observed. Surprisingly, only the lowest measured rSO_2_ values were used in their analysis, introducing a bias caused by suboptimal measurement. It remains unclear how the authors processed rSO_2_ values <15% in their results because the NIRS device used does not measure below rSO_2_ of 15% (Invos™ 5100). As the study design is fundamentally different and the measuring device (NIRS) is different, comparison with our study is not possible.

This study has several limitations. Firstly, it is a small, observational, single-center study. Larger, multicenter studies are necessary to confirm current results. Secondly, the physician of the emergency medical team was not blinded to the rSO_2_ values, although there are no indications that the rSO_2_ values were used in the decision to cease or continue resuscitation efforts. Moreover, the emergency physician did not receive any information about interpretation of rSO_2_ data. Thirdly, despite the fact that we used ROSC >20 minutes as the endpoint, only nine patients survived until arrival at the ICU and eventually only four patients survived until hospital discharge. Larger studies are necessary to compare rSO_2_ values during ALS between survivors to hospital discharge and nonsurvivors (neurological outcome). Fourthly, we could not compare our findings with end-tidal capnography, which is currently the only monitoring tool generally used during CPR, due to lack of end-tidal carbon dioxide data. Finally, none of the patients in the no ROSC group underwent an autopsy, and therefore we can only presume an arrest of cardiac origin.

## Conclusion

We found a significant difference in increase of rSO_2_ during ALS between patients who achieved ROSC and those who did not, even before ROSC was achieved. Our findings suggest that rSO_2_ could become a surrogate marker of systemic oxygenation because a significant increase in rSO_2_ was observed before ROSC. Cerebral oximetry could have the potential to guide treatment and decision-making during cardiopulmonary resuscitation, possibly together with capnography. Currently, no initial rSO_2_ or gradient cutoff value can be defined to predict ROSC. This will remain particularly difficult as high specificity is warranted for such a cutoff value, and observed rSO_2_ values vary within a wide range. Although there is no doubt that in the clinical (pre) hospital setting a tool to quickly determine whether a patient will achieve ROSC would prove invaluable, these results are promising – but there is still a way to go.

## Key messages

- Limited information and monitoring possibilities are available during ALS in the OCHA setting.
- Patients who achieve ROSC have a statistically significant higher increase in cerebral saturation from start of measurement until 2 minutes before ROSC or until end of CPR efforts.
- Further research is necessary in larger patient groups to explore the monitoring possibilities to guide CPR efforts and to correlate cerebral saturation values with neurological outcome.

## Abbreviations

ALS: advanced life support
CPR: cardiopulmonary resuscitation
NIRS: near-infrared spectroscopy
OHCA: out-of hospital cardiac arrest
ROSC: return of spontaneous circulation
rSO_2_: cerebral oxygen saturation
